# Role of 3-Mercaptopyruvate Sulfurtransferase in the Regulation of Proliferation, Migration, and Bioenergetics in Murine Colon Cancer Cells

**DOI:** 10.3390/biom10030447

**Published:** 2020-03-13

**Authors:** Fiona Augsburger, Elisa B. Randi, Mathieu Jendly, Kelly Ascencao, Nahzli Dilek, Csaba Szabo

**Affiliations:** Chair of Pharmacology, Section of Medicine, University of Fribourg, 1700 Fribourg, Switzerland; fiona.augsburger@unifr.ch (F.A.); elisa.randi@unifr.ch (E.B.R.); mathieu.jendly@unifr.ch (M.J.); kelly.ascencao@unifr.ch (K.A.); nahzli.dilek@unifr.ch (N.D.)

**Keywords:** hydrogen sulfide, bioenergetics, gasotransmitters, nitric oxide, proliferation, migration, mitochondria

## Abstract

3-mercaptopyruvate sulfurtransferase (3-MST) has emerged as one of the significant sources of biologically active sulfur species in various mammalian cells. The current study was designed to investigate the functional role of 3-MST’s catalytic activity in the murine colon cancer cell line CT26. The novel pharmacological 3-MST inhibitor HMPSNE was used to assess cancer cell proliferation, migration and bioenergetics in vitro. Methods included measurements of cell viability (MTT and LDH assays), cell proliferation and in vitro wound healing (IncuCyte) and cellular bioenergetics (Seahorse extracellular flux analysis). 3-MST expression was detected by Western blotting; H_2_S production was measured by the fluorescent dye AzMC. The results show that CT26 cells express 3-MST protein and mRNA, as well as several enzymes involved in H_2_S degradation (TST, ETHE1). Pharmacological inhibition of 3-MST concentration-dependently suppressed H_2_S production and, at 100 and 300 µM, attenuated CT26 proliferation and migration. HMPSNE exerted a bell-shaped effect on several cellular bioenergetic parameters related to oxidative phosphorylation, while other bioenergetic parameters were either unaffected or inhibited at the highest concentration of the inhibitor tested (300 µM). In contrast to 3-MST, the expression of CBS (another H_2_S producing enzyme which has been previously implicated in the regulation of various biological parameters in other tumor cells) was not detectable in CT26 cells and pharmacological inhibition of CBS exerted no significant effects on CT26 proliferation or bioenergetics. In summary, 3-MST catalytic activity significantly contributes to the regulation of cellular proliferation, migration and bioenergetics in CT26 murine colon cancer cells. The current studies identify 3-MST as the principal source of biologically active H_2_S in this cell line.

## 1. Introduction

The endogenous gaseous transmitter hydrogen sulfide (H_2_S) has been implicated in multiple regulatory processes in mammalian cells in health and disease. In mammalian cells, there are at least three significant enzymatic sources of H_2_S: Cystathionine-beta-synthase (CBS), cystathionine-gamma-lyase (CSE) and 3-mercaptopyruvate sulfurtransferase (3-MST) [1,2,3,4]. In the last decade, a novel concept emerged in the field of cancer biology, demonstrating that various cancer cells can increase their endogenous H_2_S levels and utilize H_2_S in an autocrine and paracrine manner to promote cell proliferation, cytoprotective signalling, cellular bioenergetics, and angiogenesis. Depending on the type of cancer, CBS, CSE and/or 3-MST have been implicated as enzymatic sources of H_2_S [3,4,5,6,7]. From these three enzymes, the function of the 3-MST system in cancer cells is the least understood.

In the current study, using the murine colon cancer cell line CT26 (in which, as the data presented in the current report indicate, 3-MST is the predominant endogenous enzymatic source of H_2_S), we have assessed the role of 3-MST catalytic activity in fundamental cellular functions in vitro. To this aim, we have utilized a newly discovered pharmacological 3-MST inhibitor, 2-[(4-hydroxy-6-methylpyrimidin-2-yl)sulfanyl]-1-(naphthalen-1-yl)ethan-1-one (HMPSNE) [8]. The data presented in the current article indicate that in CT26 cells, the 3-MST/H_2_S system significantly contributes to the maintenance of cell proliferation, migration and cellular bioenergetics.

## 2. Materials and Methods 

### 2.1. Cell Culture

The CT26 mouse colon carcinoma cell line (ATCC; American Type Culture Collection, Manassas, VA, USA) was maintained in DMEM containing high glucose, GlutaMAX™ Supplement and pyruvate (Gibco, ThermoFisher, Basel, Switzerland), supplemented with 10% FBS (Hyclone, Pittsburgh, PA, USA), 100 units/mL of penicillin and 100 µg/mL of streptomycin.

For each experiment involving CT26, cells were detached from culture flask by incubating 5 min at 37 °C with trypsin after rinsing with PBS.

### 2.2. Western Blotting 

CT26 cells were centrifuged 5 min at 400× g in order to resuspend the pellet in RIPA Lysis and Extraction Buffer (Thermo Scientific, Waltham, MA, USA) complemented with Halt™ Protease and Phosphatase Inhibitor Cocktail (Thermo Scientific) just before use. Protein concentrations were determined by measuring the absorbance in Pierce™ Coomassie Plus (Bradford) Assay Reagent (Thermo Scientific) using Infinite 200 Pro reader (Tecan, Männedof, Switzerland). Samples were prepared for gel electrophoresis in Bolt™ LDS Sample Buffer (4X) (Invitrogen) and Bolt™ Reducing Agent (10X) (Invitrogen, Thermo Scientific) according to manufacturer’s instructions, loaded in Bolt™ 4%–12% Bis-Tris Plus Gels (Invitrogen, Thermo Scientific) and ran at constant 120 V. Proteins were transferred to nitrocellulose membranes by dry transfer using the iBlot™ 2 Device and Transfer Stacks (Invitrogen). The membrane blocking was done in TBS/0.1% Tween/5% BSA Buffer (TBST/5% BSA). Primary antibodies were purchased from Abcam (Cambridge, UK): anti-CBS 1/500 (ab135626), anti-3-MST 1/500 (ab85377), anti-CSE 1/500 (ab151769), anti-ETHE-1 1/1000 (ab174302), anti-TST 1/400 (ab231248). The mouse anti-beta-Actin antibody 1/10,000 was obtained from Sigma-Aldrich. The incubation was done overnight at 4 °C under agitation, then the membranes were washed with TBST, incubated for 1 h at RT with the secondary antibodies anti-rabbit IgG or anti-mouse IgG, HRP-linked antibody (CST, Danvers, MA, USA) diluted 1/5000 in TBST/5% Milk. The detection was performed with Amersham ECL™ Prime Western Blotting Detection Reagent (GE Healthcare, Pittsburgh, PA, USA) and the chemiluminescence was detected with Azure Imaging System 300 (Azure Biosystems, Dublin, CA, USA). The process was applied to five different passages of the CT26 cell line.

### 2.3. RNA Isolation, RT, and RT-qPCR

CT26 cells were centrifuged 5 min at 400× g in order to resuspend the pellet in lysis buffer and isolate RNA using NucleoSpin RNA Plus kit (Macherey-Nagel, Düren, Germany). Nucleic acid concentration of samples was determined with NanoDrop spectrophotometer, and 3 µg of RNA was used for the reverse transcription using M-MLV Reverse Transcriptase (TermoFisher). RT-qPCR assays were performed with the following mouse primers purchased from Microsynth: CBS (Fw: 5′-CCT ATG AGG TGG AAG GGA TT-3′; Rev: 5′-TGT AGT TCC GCA CAG AGT CA-3′), 3-MST (Fw: 5′-ACA TCC CGG CTC AGT AAA CA-3′; Rev: 5′-TGT GTC CTT CAC AGG GTC TTC-3′), CSE (Fw: 5′-TGC CTC ACC CCA TTT CAT CT-3′; Rev: 5′-GAG TAA ACT GGG TGA GGG CT-3′), ETHE-1 (Fw: 5′-AGC TGC ACC TAT ACC TAC CTT C-3′; Rev: 5′-AGC TCC TTA ATC AAC TGA GCA TC-3′), TST (Fw: 5′-GGA GCC CGG ATA TAG TAG GAC TAG A-3′; Rev: 5′-TTC GTC AGG AAG TCC ATG AA-3′), and 18sRNA (Fw: 5′-GTA GTT CCG ACC ATA AAC GA-3′; Rev: 5′-TCA ATC TGT CAA TCC TGT CC-3′). SensiFAST Probe Hi-ROX Kit (Bioline, 82020, Memphis, TN, USA) was used for primers amplification with StepOnePlus Real-Time PCR System. The process was applied to four different passages of CT26 cell line.

### 2.4. Detection of H_2_S Production

Human recombinant 3-MST was produced in an *E. coli* expression system and purified by GenScript (USA). The catalytic activity of 3-MST was tested in presence of the 3-MST inhibitor [8] 2-[(4-hydroxy-6-methylpyrimidin-2-yl)sulfanyl]-1-(naphthalen-1-yl)ethan-1-one (HMPSNE) (MolPort, Riga, Latvia) in black 96-well plates. HMPSNE was diluted in reaction buffer 50 mM Tris-HCl, pH 8 from a 500 mM stock solution in 100% DMSO. First, 3 µg/well of recombinant 3-MST were incubated 1 h at 37 °C with indicated concentrations of HMPSNE in a total volume of 100 µl. After adding HMPSNE to yield various final concentrations, the 3-MST substrate 3-mercaptopyruvate (Sigma-Aldrich, St. Louis, MI, USA) was added for a final concentration of 2 mM and the H_2_S sensitive fluorescent probe 7-azido-4-methylcoumarin (AzMC) [9,10] (Sigma-Aldrich) for a final concentration of 10 µM. Fluorescence was immediately measured in kinetic mode for 2 h at 37 °C with an Infinite 200 Pro reader (Tecan), with excitation and emission wavelengths of 365 nm and 450 nm, respectively. The final concentration of DMSO was kept constant at 0.2% in all conditions. The assay was repeated three times in triplicates. Data analysis was performed after background H_2_S fluorescence removal, which is known to be produced by the spontaneous release of H_2_S from 3-mercaptopyruvate [11]. The IC_50_ of the inhibitor was calculated using Graphpad Prism nonlinear fitting curve function on data points recorded at 1 h.

The activity of 3-MST in presence of various concentrations of HMPSNE was also tested in CT26 cells homogenates. CT26 cells were centrifuged 5 min at 400× g in order to resuspend the pellet in lysis buffer 150 mM NaCl and 50 mM Tris-HCl, pH 8 containing 1% NP40 and Halt™ Protease and Phosphatase Inhibitor Cocktail (Thermo Scientific) added just before use. Samples were sonicated with ultrasound probe three times a cycle of 5 s on and 5 s off at 70% amplitude and kept on ice for 30 min. The protein concentrations were measured as described above. HMPSNE dilutions were prepared as above and incubated 24 h at 37 °C with 100 µg/well of proteins from CT26 homogenates in black 96-well plate. After treating the homogenate with HMPSNE, 3-mercaptopyruvate was added for a final concentration of 500 µM and AzMC for a final concentration of 10 µM. Fluorescence measurement and analysis were performed as above.

The same AzMC probe was used to measure H_2_S in live CT26 cells as previously described for various other cell types [9,10]. Cells were seeded in sterile black 96-well plate with optical bottom at 20,000 cells/well in 100 µl of complete culture medium. After 1h incubation at 37 °C and 5% CO_2_ to let the cells attach, supernatant was replaced by medium containing various concentrations of HMPSNE as indicated. After 3 h incubation, supernatant was replaced by 1 mM AzMC prepared in HBSS 1X supplemented with glucose (Gibco), and the cells were incubated one more hour. Pictures were taken using Olympus CKX53 inverted microscope and fluorescence intensity per cell was quantitated by the ImageJ program (NIH, Bethesda, MD, USA).

### 2.5. Growth Monitoring, Viability and Metabolic Assay

CT26 cells were seeded in sterile transparent 96-well plate at 5,000 cells/well in 100 µl of complete culture medium. Various concentrations of HMPSNE and aminooxyacetate hemihydrochloride (AOAA) (Sigma-Aldrich) were added as indicated. The plate was placed in an IncuCyte Live Cell Analysis device (20x objective) (Essen Bioscience, Herforthshire, UK) and the confluence was recorded every hour by phase contrast scanning for 48 h at 37 °C and 5% CO_2_. Then, 50 µl of each well’s supernatant was transferred in another plate to test lactate dehydrogenase (LDH) activity, an indicator of cell necrosis. The remaining medium was discarded and 50 µl/well of serum-free medium was added on top of the cells to test mitochondrial activity with 3-(4,5-dimethylthiazol-2-yl)-2,5-diphenyltetrazolium bromide (MTT). The LDH assay was performed as described [12] using the Pierce LDH Cytotoxicity Assay Kit (Thermo Scientific). Briefly, LDH reaction mixture was prepared according to manufacturer’s instructions, and 50 µl/well were added to the supernatants. The plate was incubated 30 min at RT protected from light, and the reaction was stopped with 50 µl/well of Stop Solution from the kit. The plate was shaken in an orbital way for 60 s by Infinite 200 Pro reader (Tecan) before measuring the absorbance at 490 nm and 680 nm (background). The MTT assay was performed as described [12] using MTT Cell Proliferation Assay Kit (Abcam). The cells in serum-free medium were incubated 3 h at 37 °C and 5% CO_2_ with 50 µl/well of MTT reagent. Formazan produced by cells with active metabolism was solubilized in 150 µl/well of MTT solvent by mixing well and shaking in orbital way 15 min at RT protected from light. The absorbance was measured at 590 nm using Infinite 200 Pro reader (Tecan). The LDH and MTT assays were repeated at least 4 times in triplicates per compound’s concentration.

The LDH and MTT assays were also performed with CT26 cells seeded at 10,000 cells/well and incubated with HMPSNE or AOAA at indicated concentrations for 24 h at 37 °C and 5% CO_2_.

### 2.6. Migration Assay

CT26 cells were seeded in sterile transparent 96-well plate at 50,000 cells/well in 100 µl of complete culture medium and incubated 24 h at 37 °C and 5% CO_2_. The WoundMaker from Essen BioScience was used to create homogeneous wide wounds in the cell monolayer of each well. Then the culture medium was carefully replaced with indicated HMPSNE serial dilutions in complete medium containing IncuCyte Cytotox Green Reagent (Essen BioScience) for counting dead cells. The plate was placed in IncuCyte device (10x objective) and the confluence was recorded every 2 h by both phase contrast and fluorescence scanning for 48 h at 37 °C and 5% CO_2_. Images were analyzed using the Incucyte ZOOM software.

### 2.7. Determination of Cellular Bioenergetics

Cellular bioenergetics was measured by the Extracellular Flux Analysis method as described for various other cell types [10,13]. Cells (15,000/well) were seeded on cell culture microplates and treated with HMPSNE (10, 100, or 300 µM) or its vehicle for 16 h. For analysis of mitochondrial respiration, cells were washed twice with DMEM (pH 7.4) supplemented with L-glutamine (2 mM, Gibco), sodium pyruvate (1 mM, Sigma) and glucose (10 mM, Sigma). After 1 h incubation at 37 °C in CO_2_-free incubator, the oxygen consumption rate (OCR) after oligomycin (1 µM) was used to estimate the rate of ATP production. In addition, carbonyl cyanide-4-trifluoromethoxy phenylhydrazone (FCCP, 1.2 µM) was used to estimate the maximal mitochondrial respiratory capacity. The flux of electrons through complex III and I was blocked with antimycin A (0.5 µM) and rotenone (0.5 µM), respectively; any residual activity in the presence of these inhibitors was assessed as non-mitochondrial OCR.

For the analysis of glycolytic parameters, cells were treated with HMPSNE as above, washed twice with phenol red-free DMEM (pH 7.4) containing L-glutamine (2 mM), sodium pyruvate (1 mM), glucose (10 mM) and HEPES (5 mM). After incubation at 37 °C in CO_2_-free incubator for 1 h, proton efflux rate (PER) from basal and compensatory glycolysis was measured. Rotenone (0.5 µM) and antimycin A (0.5 µM) were used to estimate mitochondrion-associated acidification. Moreover, 2-deoxy-D-glucose (50 mM) was used to stop the glycolytic acidification of the cells.

The specific activity of Complex IV in permeabilized cells was measured by the Extracellular Flux Analysis method as described [10,14]. Briefly, cells were washed twice with Mannitol-Sucrose-BSA (MAS-BSA) buffer pH 7.2 (70 mM sucrose, 220 mM mannitol, 10 mM KH_2_PO_4_, 5 mM MgCl_2_, 2 mM HEPES, 1 mM EGTA, and 4 mg/mL fatty acid-free BSA). OCR was measured twice at steady state and following each port injection, which were prepared as follows: A) XF PMP permeabilizer (1 nM) (Agilent Technologies)/tetramethyl-p-phenylene diamine (TMPD, 0.5 mM)/ascorbate (2 mM)/FCCP (1 µM)/ADP (1 mM); B) oligomycin (1 μg/mL); C) sodium azide (NaN_3_, 20 mM).

### 2.8. Statistical Analysis

Data are presented as representative blots or mean ± SEM values of experiments performed on at least n = 3 experimental days. ANOVA followed by post-hoc analysis by Dunnett’s multiple comparisons test was used to analyze the numerical data. A *p* < 0.05 was considered statistically significant.

## 3. Results

### 3.1. Expression Analysis of 3-MST and the other H_2_S Pathway Enzymes in CT26 Cells

**Figure 1:**
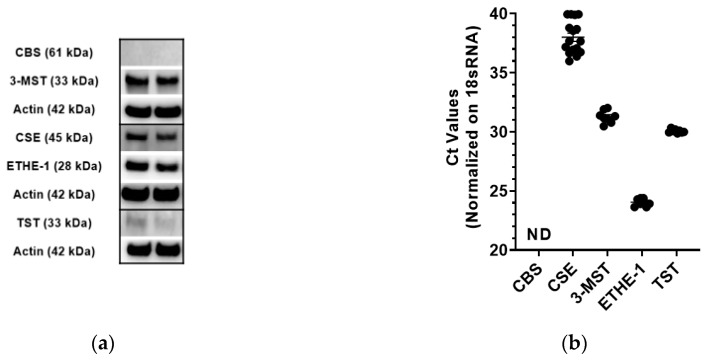
Qualitative detection of H_2_S pathway enzymes in CT26 cells at RNA and protein levels: (**a**) Representative Western blot of cystathionine-beta-synthase (CBS), 3-mercaptopyruvate sulfurtransferase (3-MST), cystathionine-gamma-lyase (CSE), ETHE-1, and TST in mouse colon carcinoma CT26 cell lysates; (**b**) Ct values from RT-qPCR amplification curves of CT26 cDNA with primers for mouse CBS, CSE, 3-MST, ETHE-1, and TST. Ct value of 18sRNA amplification was used for normalization. Single values are given by dots and corresponding mean ± SEM by lines. ND indicates non-detectable amplification.

The expression of 5 principal enzymes related to mammalian H_2_S generation and degradation was evaluated in CT26 cells by Western blotting. Significant protein expression of 3-MST, CSE, ETHE-1, weak expression of TST, and no expression of CBS was found by Western blotting (Figure 1a). At the RNA level, 3-MST and TST were detected in significant amounts; ETHE-1 was found to be very highly expressed, CSE mRNA showed a low-level expression, while CBS mRNA was not detectable (Figure 1b).

### 3.2. Inhibition of 3-MST-Mediated H_2_S Production by HMPSNE

**Figure 2:**
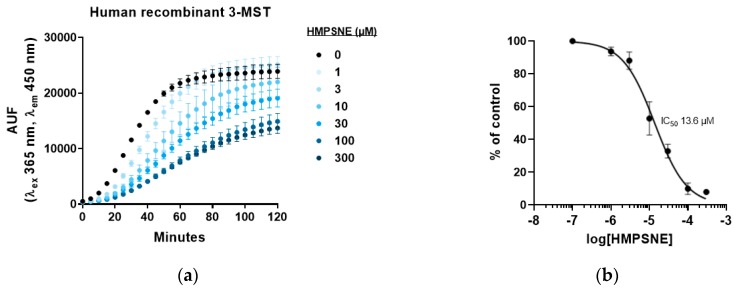
Human recombinant 3-MST activity in presence of 2-[(4-hydroxy-6-methylpyrimidin-2-yl)sulfanyl]-1-(naphthalen-1-yl)ethan-1-one (HMPSNE), measured with the fluorescent H_2_S probe 7-azido-4-methylcoumarin (AzMC): (**a**) Representative curves of H_2_S production after incubation with serial dilutions of HMPSNE, in presence of 2 mM 3-MP and 10 µM AzMC. Data are presented as mean ± SEM of three replicates; (**b**) Concentration-response of HMPSNE represented in percentage of control as mean ± SEM of three independent experiments.

The recently identified pharmacological inhibitor of 3-MST, HMPSNE [8] produced a concentration-dependent inhibition of the AzMC signal when incubated with purified human recombinant enzyme (Figure 2a), confirming that inhibition of the catalytic activity of 3-MST produces a concentration-dependent inhibition of H_2_S production. The IC_50_ of HMPSNE was calculated as 13.6 µM under our experimental conditions (Figure 2b).

**Figure 3:**
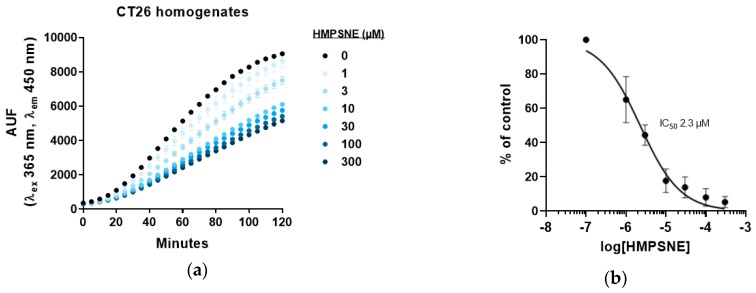
3-MST activity in CT26 homogenates measured with AzMC in presence of HMPSNE: (**a**) Representative curves of H_2_S production after incubation with serial dilutions of HMPSNE, in presence of 500 µM 3-MP and 10 µM AzMC. Data are presented as mean ± SEM of three replicates; (**b**) Concentration response of HMPSNE represented in percentage of control as mean ± SEM of three independent experiments.

The inhibition by HMPSNE was also tested on the activity of 3-MST from CT26 homogenates, which contain the murine form of the enzyme. Similar to the data obtained with human 3-MST, a concentration-dependent decrease of AzMC fluorescence was noted (Figure 3a); The IC_50_ of HMPSNE for murine 3-MST was calculated as 2.3 µM under our experimental conditions (Figure 2b).

**Figure 4:**
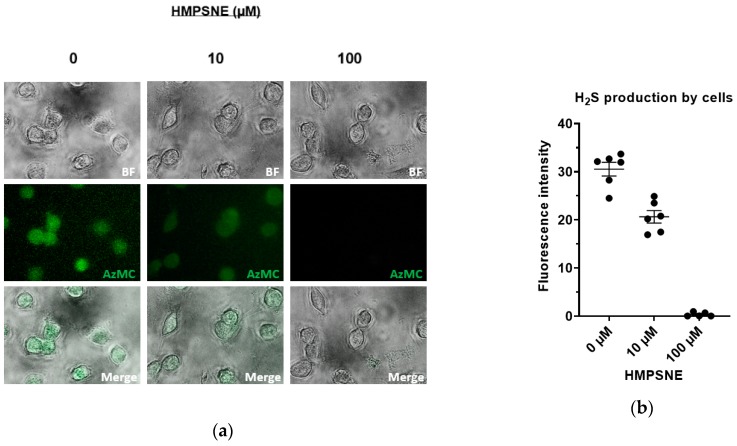
H_2_S production by live CT26 pretreated with HMPSNE: (**a**) CT26 cells were incubated 1 h with the probe AzMC after 3 h of incubation with HMPSNE at 10 µM and 100 µM or its vehicle. From top to bottom are brightfield, green channel and merged images taken with inverted microscope; (**b**) Quantification of fluorescence signal (analysis of the images was made using ImageJ). Single values are given by dots and corresponding mean ± SEM by lines.

Moreover, when H_2_S production by live CT26 cells was measured with AzMC, 10 µM HMPSNE caused a partial inhibition of the signal, while at 100 µM HMPSNE a complete inhibition of the AzMC-guided H_2_S fluorescence was found (Figure 4a,b). These data indicate that the 3-MST inhibitor has acceptable cell permeation and is capable of inhibiting its target in situ in CT26 cells (with an IC_50_ of approximately 30 µM).

### 3.3. Effect of 3-MST Inhibition on CT26 Growth, Migration, and Viability

**Figure 5:**
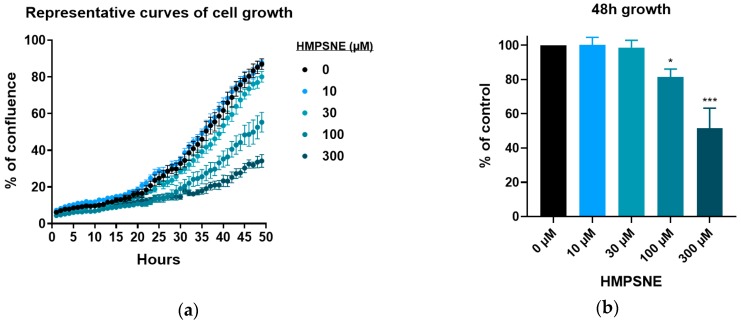
3-MST inhibition slows down proliferation of CT26: (**a**) Confluence of cells untreated or treated with HMPSNE at very low density was recorded each hour for 48 h. Data are presented as mean ± SEM of three replicates; (**b**) Cell confluence after 48 h growth shown as percentage of untreated cells. Data are presented as mean ± SEM of minimum three independent experiments and Dunnett’s multiple comparisons tests are shown vs. 0 µM (* *p* < 0.05, ** *p* < 0.01, *** *p* < 0.001).

**Figure 6:**
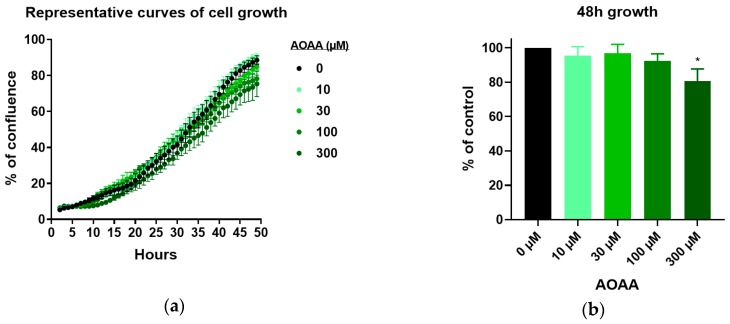
Aminooxyacetate hemihydrochloride (AOAA), (a pharmacological inhibitor of CBS) has minimal effect on CT26 proliferation: (**a**) Confluence of cells untreated or treated with AOAA at very low density was recorded each hour for 48 h. Data are presented as mean ± SEM of three replicates; (**b**) Cell confluence after 48 h growth shown as percentage of untreated cells. Data are presented as mean ± SEM of minimum three independent experiments and Dunnett’s multiple comparisons tests are shown vs. 0 µM (* *p* < 0.05).

After confirming that CT26 cells express 3-MST that is inhibited by HMPSNE, and that this results in the suppression of H_2_S production by the cells, the next aim of our study was to assess its impact on cell growth, viability and migration. CT26 cells were found to proliferate less with increasing concentrations of HMPSNE, as assessed by the IncuCyte method (Figure 5a,b). In contrast, AOAA (a compound which is widely considered a CBS inhibitor, but it in fact inhibits both CBS and CSE) did not have any major effect on proliferation (Figure 6a,b), in line with the finding that this cell line does not express detectable amounts of CBS (Figure 1). The slight inhibition of cell proliferation at 300 µM AOAA is likely represents an effect on additional pharmacological targets of AOAA; this inhibitor is known to inhibit a number of transaminases [3,15,16].

**Figure 7:**
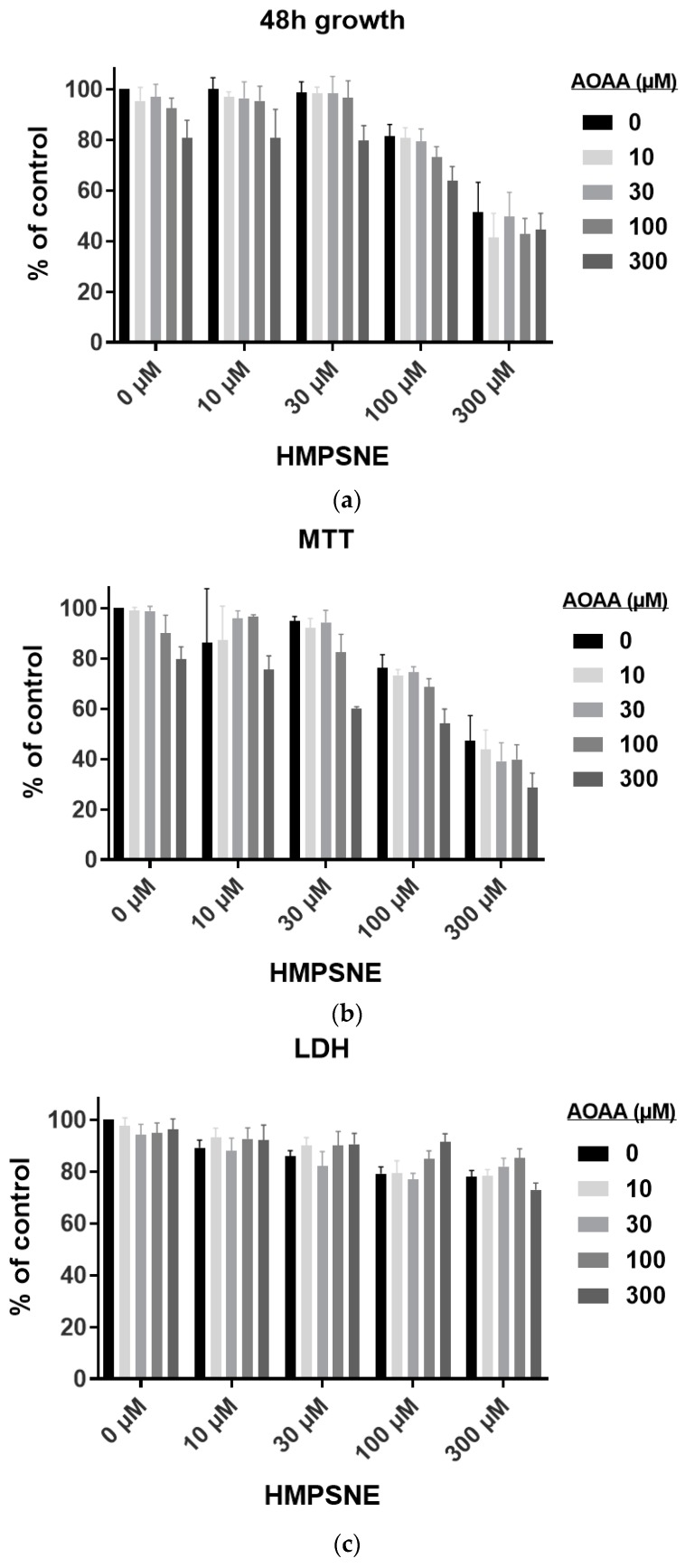
Proliferation, metabolic activity and cell death by necrosis of CT26 incubated with combination of HMPSNE and AOAA: (**a**) Cell confluence after 48 h growth shown as percentage of untreated cells; (**b**) MTT conversion by CT26 cells after 48 h growth shown as percentage of untreated cells; (**c**) lactate dehydrogenase (LDH) activity in medium of CT26 grown for 48 h shown as percentage of untreated cells. All graphs present data from at least four independent experiments.

The combination of AOAA and HMPSNE did not show any significant additive nor synergistic effect on cell proliferation (Figure 7a). These findings reinforce the conclusion that the major H_2_S-producing enzyme involved in the regulation of CT26 cell proliferation is 3-MST (and not CBS).

The findings obtained with the IncuCyte method were also reflected by measurements of MTT conversion: MTT conversion was concentration-dependently inhibited by HMPSNE (but not by AOAA) and there were no additive or synergistic effects between the two inhibitors (Figure 7b). In the concentration range used in the current study (10–300 µM) neither HMPSNE nor AOAA produced any significant increase in LDH levels in the supernatant, suggesting that neither inhibitor exerted any direct cytotoxic effects (Figure 7c).

**Figure 8:**
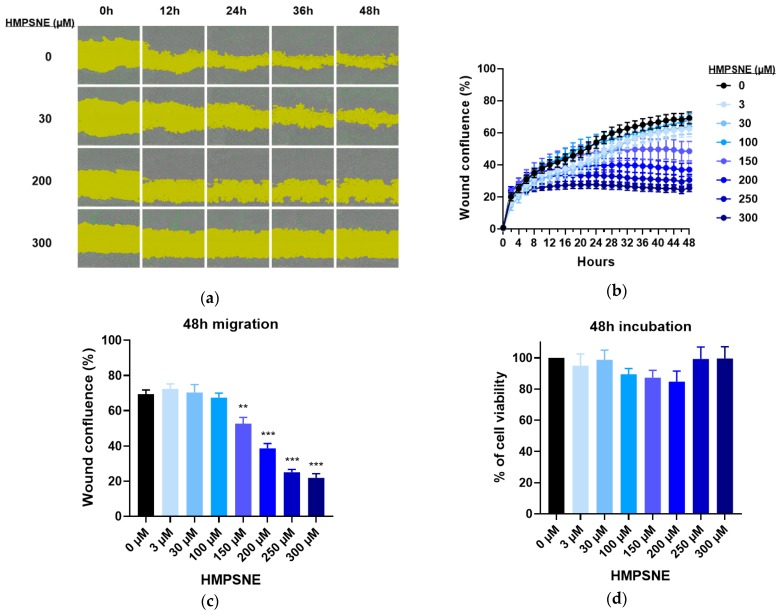
Scratch wound CT26 migration assay with 3-MST inhibitor: (**a**) Representative images taken with Incucyte 10x showing wound area (yellow) coverage by cells and real-time cell death (green) every 12h for 48h; (**b**) Representatives curves of wound confluence in percentage over time with serial dilutions of HMPSNE. Data are presented as mean ± SEM of three replicates per condition; (**c**) Wound confluence in percentage after 48 h. Data are presented as mean ± SEM of three independent replicates per condition, and Dunnett’s multiple comparisons tests are shown vs. 0 µM (* *p* < 0.05, ** *p* < 0.01, *** *p* < 0.001); (**d**) Cell viability after 48 h migration, assessed by the fluorescence of cyanine nucleic acid dye. Data are presented as mean ± SEM of three replicates.

The next sets of experiments tested the effect of 3-MST inhibition on CT26 cell migration. Wound areas were created on cell monolayers to observe their invasion over time and to evaluate changes in the migration capacity of CT26 in response to pharmacological inhibition of 3-MST (Figure 8a). Migration diminished with increasing concentrations of HMPSNE, reaching complete cell immobility at 300 µM (Figure 8b,c). However, cell death was comparable to the untreated cells for all conditions (Figure 8d), in accordance with the LDH activity measurements in supernatant from cells after 48 h proliferation (Figure 7c). Therefore 3-MST inhibition does not trigger significant necrosis or apoptosis, but it suppresses the ability of the cells to proliferate and migrate.

### 3.4. Modulation of Metabolic and Glycolysis-Related Bioenergetics by HMPSNE in CT26 Cells

**Figure 9:**
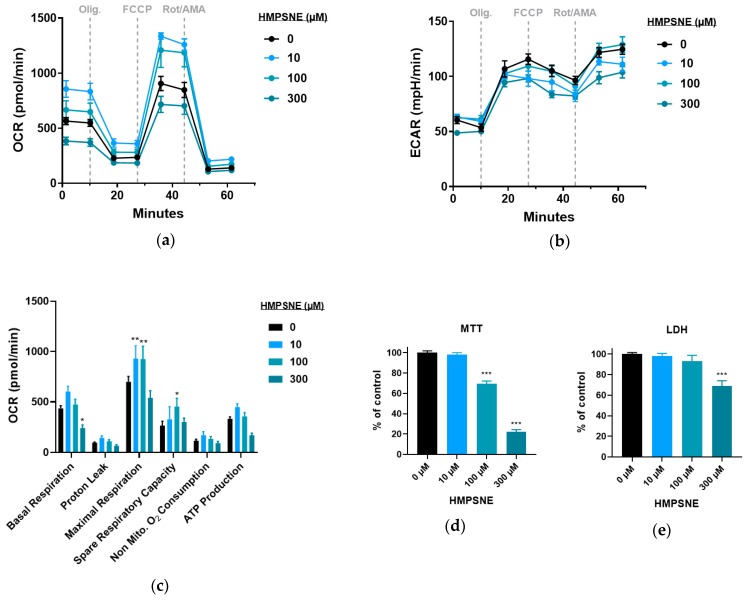
Effect of HMPSNE on oxidative phosphorylation parameters and cell viability in CT26 cells: (**a**) Oxygen consumption rate (OCR) profiles in control cells and in HMPSNE treated cells. Dashed lines indicate the addition of specific mitochondrial stressors. Results are indicated as mean ± SEM of four replicates per condition; (**b**) Representative extracellular acidification rate (ECAR) profiles in control cells and in HMPSNE treated cells. Arrows indicate the addition of specific mitochondria stressors into the media. Results are indicated as mean ± SEM of four replicates per condition; (**c**) Analysis of oxidative phosphorylation-related bioenergetics parameters. Results are indicated as mean ± SEM of three independent replicates per condition, and Dunnett’s multiple comparisons tests are shown vs. 0 µM of each parameter respectively (* *p* < 0.05, ** *p* < 0.01); (**d**) MTT conversion by CT26 cells after 24 h incubation with HMPSNE shown as percentage of untreated cells. Data are shown as mean ± SEM of three independent replicates and Dunnett’s multiple comparisons tests are shown vs. 0 µM (*** *p* < 0.001); (**e**) LDH activity in medium of CT26 after 24 h incubation with HMPSNE shown as percentage of untreated cells. Data are shown as mean ± SEM of 3 independent replicates and Dunnett’s multiple comparisons tests are shown vs. 0 µM (*** *p* < 0.001).

The effect of 3-MST inhibition on cellular bioenergetics was determined using the Seahorse extracellular flux apparatus. Oxygen consumption rate (OCR) and extracellular acidification rate (ECAR) measurements showed that CT26 cells responded to HMPSNE with a bell-shaped response: there was an enhancement of OCR at low (10 µM) and intermediate (100 µM) concentrations of HMPSNE, while we noted an inhibition at the highest concentration of HMPSNE tested (300 µM) (Figure 9a–c). In contrast to the bell-shaped effect on OCR, HMPSNE did not enhance MTT conversion at 10 µM, while at 100 and 300 µM it produced an inhibitory response (Figure 9d), without increasing the LDH signal, i.e., without inducing any detectable degree of cell necrosis (Figure 9e).

**Figure 10:**
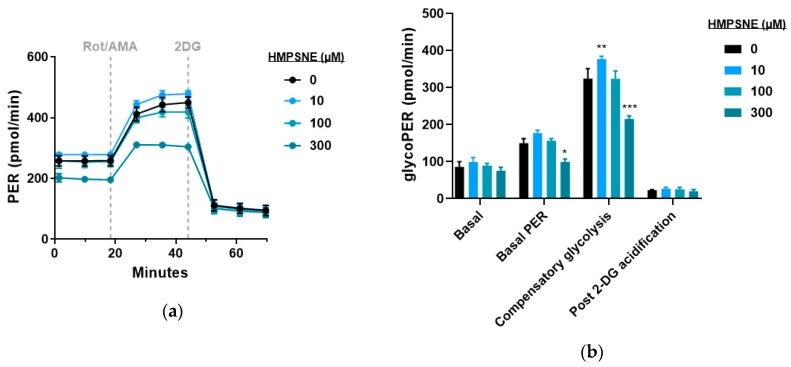
Effect of HMPSNE on glycolytic parameters in CT26 cells: (**a**) Proton efflux rate (PER) profiles in control and HMPSNE treated cells. Dashed lines indicate the addition of specific mitochondria and glycolysis stressors into the media. Results are indicated as mean ± SEM of three replicates per condition; (**b**) analysis of glycolysis-related bioenergetics parameters. Results are indicated as mean ± SEM of three independent replicates per condition, and Dunnett’s multiple comparisons tests are shown vs. 0 µM of each parameter respectively (* *p* < 0.05, ** *p* < 0.01, *** *p* < 0.001).

Proton efflux rate was unaffected with the lower concentrations of 3-MST inhibitor tested, while it was inhibited at 300 µM (Figure 10a). CT26 glycolytic parameters were affected in a bell-shaped manner (Figure 10b).

**Figure 11:**
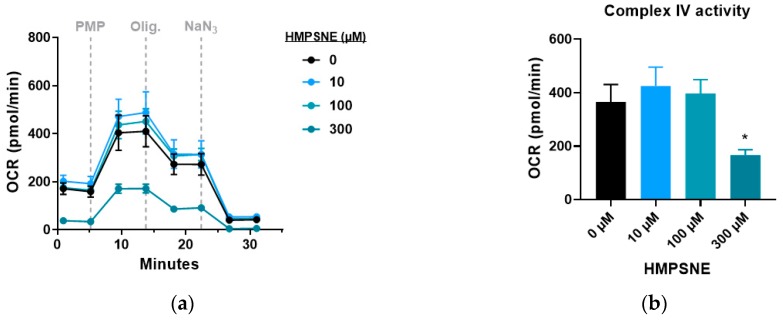
Effect of HMPSNE on Complex IV activity in CT26 cells: (**a**) Proton efflux rate (PER) profiles in control cells and HMPSNE treated cells. Dashed lines indicate the addition of specific mitochondria stressors into the media. Results are indicated as mean ± SEM of three replicates per condition; (**b**) Analysis of Complex IV activity calculated by the difference between maximal and minimal OCR. Results are indicated as mean ± SEM of three independent replicates per condition, and unpaired t tests are shown vs. 0 µM (* *p* < 0.05, ** *p* < 0.01, *** *p* < 0.001).

When examining specific Complex IV activity, HPMSNE did not significantly affect this parameter at 10 or 100 µM (although a tendency for an increase was, in fact, noted, in line with the known inhibitory effect of H_2_S on Complex IV activity [15,16]), while at the highest concentration tested (300 µM) the inhibitor suppressed this parameter (Figure 11a,b).

**Figure 12:**
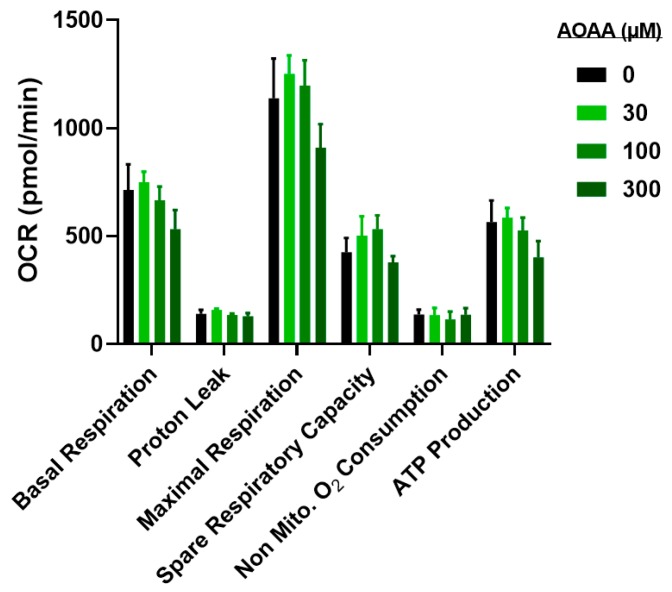
Effect of AOAA on bioenergetics parameters and cell viability in CT26 cells: Analysis of oxidative phosphorylation-related bioenergetics parameters. Results are indicated as mean ± SEM of three independent replicates per condition and corresponding Dunnett’s multiple comparisons tests vs. 0 µM of each parameter respectively are not significant.

In line with the lack of significant effect of the CBS inhibitor AOAA on various functional parameters (see above), the inhibitor also failed to exert significant effects on the extracellular flux analysis parameters (Figure 12).

## 4. Discussion

3-MST (which is often referred to as the “third” H_2_S-producing enzyme—after CBS and CSE) is part of the cysteine catabolism pathway and utilizes 3-mercaptopyruvate as a substrate [4]. 3-MST works in tandem with cysteine aminotransferase activity, which generates 3-mercaptopyruvate from cysteine. Although 3-MST is often referred as a “mitochondrial enzyme” it should be noted that the enzyme has both cytosolic and mitochondrial localizations in all cells where this has been evaluated so far [3,4]. The expression of 3-MST, as well as its biochemical properties have been characterized extensively (reviewed in [4,17,18]). The mechanism of 3-MST-mediated H_2_S production involves the transfer of 3-mercaptopyruvate’s sulfur atom to a nucleophilic cysteine (Cys247) in its active site; subsequently, the protein persulfide generates H_2_S through the action of various intracellular reductants (for example, dihydrolipoic acid or thioredoxin).

Most parenchymal cells contain 3-MST, and the enzyme is considered a significant endogenous biological source of H_2_S, as well as of biologically active reactive sulfur species (polysulfides) [18]. It should be also mentioned that 3-mercaptopyruvate, in addition to acting as a substrate of 3-MST, can also produce H_2_S spontaneously (i.e., in solution, in the absence of any cellular components or enzymes) [11,19,20].

The potential role of 3-MST in the biology of cancer cells has recently been reviewed by us [7]. An early study, focusing on the expression of 3-MST in cancer cells was performed by Wrobel’s group and was found to detect significant 3-MST expression and enzymatic activity in several different human neoplastic cell lines (including U373 astrocytoma, SH-SY5Y neuroblastoma, and the melanoma lines A375 and WM35) [6,21]. There are many additional cancer cell lines where the presence of 3-MST has been demonstrated; these include the glioblastoma-astrocytoma cell line U-87 [6], the hepatoma lines Hepa1c17 and HepG2 [22,23], various human colon cancer cell lines (HCT116, HT-29, LoVo,) [24,25,26], the human lung adenocarcinoma line A549 [27,28], the RCC4 renal cell carcinoma line [29], several different urothelial cancer cell lines [30,31] and several melanoma cell lines (PES 43, A375, Sk-Mel-5, Sk-Mel-28) [32]. The results of the current study add the murine colon cancer cell line CT26 to the list of tumor cells that exhibit high 3-MST expression.

3-MST expression was also clearly demonstrated in many different primary tumor tissues, including human brain gliomas [33], human melanoma specimens [34], human colon cancer resections [26], human lung carcinoma resections [27], human urothelial cell carcinoma of the bladder resections [30,31], human oral squamous cell carcinoma resections [35], and oral adenoid cystic carcinoma resections [36]. In many (but not all) of these studies, the expression of 3-MST tended to increase with higher grades of the malignancy. In OncoBase (http://www.oncobase.biols.ac.cn/), an integrated database for annotating somatic mutations in different cancer types, 289 mutations were described for 3-MST gene in 36 cancers retrieved from The Cancer Genome Atlas (TGCA), although the nature of these mutations with respect to the functionality of 3-MST protein has not yet been elucidated.

Although the potential functional role of 3-MST in various cancer cells is indirectly supported by the above observations, the large majority of the prior body of work on 3-MST and cancer was based on comparative studies, correlational studies, or expression profiling (rather than the type of direct studies presented in the current article, targeting the catalytic activity of the enzyme with a pharmacological inhibitor). Nevertheless, there were several intriguing observations and suggestions in the literature regarding 3-MST. For instance, 3-MST was found to be upregulated in multidrug-resistant and stem cell-like cancer cell lines [23,25]. This finding—together with the various emerging biological roles of H_2_S in cancer cells in the promotion of cell proliferation, migration and anticancer therapy resistance [reviewed in 5]—suggests the potential functional importance of this enzyme in malignancies. These observations suggested (but certainly did not prove) that inhibition of 3-MST may exert some potential anticancer effects in vitro or in vivo.

The lack of direct studies interrogating the functional role of 3-MST was primarily due to the fact that until recently, 3-MST pharmacological inhibitors of sufficient potency and selectivity were not available. Several recent studies, conducted in hepatoma cell lines and in lung carcinoma cell lines suggested that silencing of 3-MST suppresses cancer cell bioenergetics and/or proliferation [7,22]. However, in these studies, the degree of 3-MST silencing was incomplete and the corresponding partial effects were difficult to interpret. Moreover, the studies based on 3-MST expression silencing did not distinguish between functional effects due to the inhibition of the catalytic function of 3-MST (i.e., suppression of H_2_S and polysulfide generation) vs. other catalytic functions of 3-MST, vs. the lack of the enzyme itself (i.e., the inhibition of scaffolding effects of the enzyme or other roles that require the physical presence of the enzyme). This situation changed with the work of Hanaoka and colleagues, who have conducted a screening campaign and identified several novel inhibitors of the enzyme, with one of them (HMPSNE, used in our current experiments) exhibiting the best potency and selectivity profile (for 3-MST, as opposed to CBS or CSE) [8].

In the current study, we were able to confirm the inhibitory effect of HMPSNE both in human and in murine 3-MST. The IC_50_ values were not identical, which could be attributed either to differences between the human vs. murine enzyme (which have approximately 85% homology) and/or to the difference between the assay conditions (recombinant enzyme vs. cell homogenate for the human vs. murine 3-MST, respectively). The inhibitory effect of HMPSNE on 3-MST in situ was also directly confirmed using a live cell imaging assay; in this assay the IC_50_ was slightly higher, with full inhibition of detectable H_2_S production obtained at 100 µM of the inhibitor.

In various cancer cells, endogenously produced H_2_S—from different enzymatic sources: CBS, CSE and/or 3-MST, depending on the cancer type [5]—has been shown to contribute to the maintenance of cancer cell growth, invasiveness, anticancer drug resistance, and tumor angiogenesis. In particular in human colon cancer, the most significant upregulation in tumor tissue (compared to surrounding tissue) was noted in CBS, with a trend for increase for 3-MST [26]. Likewise, in many human colon cancer cell lines, CBS was found to be highly expressed, and genetic silencing or pharmacological inhibition of CBS exerted antitumor effects in vitro and in vivo [24,25,26,37,38,39,40,41] while forced expression of CBS into non-transformed human epithelial cells increased their growth in situ (but did not render them metastatic) [42]. In contrast, in the current, murine colon cancer cell line, CBS (mRNA or protein) was undetectable (and, accordingly, the CBS inhibitor AOAA was without significant biological effects), while 3-MST was highly expressed. This finding highlights species differences between the regulation of H_2_S biosynthesis in colon cancer; the current study also identifies the CT26 cells as a potentially useful model to investigate 3-MST-related biological processes in the complete absence of CBS.

The third major H_2_S-producing enzyme, CSE was also expressed in CT26 cells (Figure 1). Several prior studies demonstrate that in some tumor cells CSE-derived H_2_S is involved in the maintenance of homeostasis [5]. For instance, in some human colon cancer cell lines (SW480 and RKO cells) CSE is expressed and its silencing or its pharmacological inhibition suppresses cell proliferation and migration [43], while in other colon cancer lines (e.g., HCT116), CSE is also expressed, but its silencing does not affect cell proliferation or bioenergetics [24]. Although AOAA is commonly referred to in the literature as a “CBS inhibitor”, this compound, in fact, inhibits both CBS and CSE [44]. However, since in the current study AOAA does not affect the functions we have studied, we must conclude that—at least in the current cell line—neither CSE nor CBS play a significant functional role. Moreover, at least theoretically, AOAA may also inhibit the 3-MST pathway, because it inhibits CAT, the enzyme involved in the generation of 3-mercaptopyruvate from cysteine [15,16]. However, the current findings indicate that this mechanism does not appear to be significant in CT26 cells, because AOAA (at least in the concentration range used) did not affect any of the parameters investigated.

The effects of HMPSNE on CT26 cell function were found to be consistent with the previously demonstrated roles of endogenously produced H_2_S in various tumor cells [5]; the 3-MST inhibitor induced a concentration-dependent inhibition of cell proliferation and cell migration. These effects were noted in the concentration range of 30–300 µM, which is consistent with the concentrations that inhibit H_2_S production according to our live cell imaging assay. Although 100 µM HMPSNE was already sufficient to fully suppress H_2_S production (at least, to the detection limit of our live cell imaging assay that relies on the fluorescent dye AzMC), the increase of HMPSNE’s concentration from 100 to 300 µM produced further inhibitory effects. These effects may be either related to additional pharmacological actions of HMPSNE (unrelated to 3-MST inhibition) and/or may indicate that a small amount of H_2_S may be sufficient to exert various biological effects, and a near-complete or complete inhibition of all 3-MST-derived H_2_S production is necessary to exert full biological responses. The findings of the current study are in line with a recent paper testing the effect of HMPSNE in vascular endothelial cells, where the 3-MST inhibitor, in a similar concentration range of 30–100 µM suppressed cell proliferation, migration and—most significantly—the formation of tube-like structures [45]. We have also noted that while at 100 µM, the inhibitory effect of HMPSNE on MTT conversion was comparable at 24 h and 48 h, at 300 µM the inhibition at 24 h seemed to be more pronounced than at 48 h (Figure 7b vs. Figure 9d). It is possible that at the highest concentration used (300 µM), HMPSNE exerts additional pharmacological effects on cellular targets other than 3-MST and this may possibly contribute to this difference.

H_2_S exerts a bell-shaped effect (stimulation at lower concentrations and inhibition at higher concentrations) on aerobic bioenergetic parameters (mitochondrial electron transport) and on glycolytic parameters (i.e., anaerobic bioenergetic parameters), and this is produced from actions on multiple molecular targets [15,16]. The stimulatory effects on mitochondrial function include direct electron donation [15,16], inhibition of mitochondrial cAMP phosphodiesterases [44] and stimulation of ATP synthase [46], while the inhibitory effect is primarily attributed to inhibition of Complex IV (cytochrome C oxidase), although, at higher H_2_S concentrations, ATP synthase can also be inhibited by H_2_S [47]. The effects of HMPSNE on CT26 bioenergetic parameters also tended to show a bell-shaped concentration-response (a stimulatory effect at lower concentrations, e.g., 10 µM) and an inhibitory effect at the top end of the concentration-response curve (300 µM). These findings are not entirely consistent with the functional effects noted: At 10 µM HMPSNE we did not observe any effect on cell proliferation or migration, even though at this concentration CT26 bioenergetic parameters were increased. Also, at 100 µM HMPSNE (a concentration where cell proliferation or migration were inhibited), most of the cellular bioenergetic parameters were comparable to the vehicle-control, indicating that the HMPSNE-induced functional effects are not directly or not necessarily linked to the HMPSNE-induced bioenergetic alterations in CT26 cells. Nevertheless, the suppression of cellular bioenergetics at 300 µM and the suppression of cell proliferation or migration seen at 300 µM are in line with each other. It remains to be further clarified how the effects of HMPSNE are related to the bell-shaped concentration-response of H_2_S in mitochondria. Since 3-MST is partially mitochondrial, it is conceivable that it produces H_2_S in the vicinity of Complex IV, and the blockade of this inhibitory action may yield the stimulation observed at 10 µM HMPSNE. (There are, in fact, several prior examples of endogenously produced H_2_S suppressing, rather than increasing mitochondrial function: for instance, CSE-derived H_2_S does so in normal mouse liver [48] and CBS-derived H_2_S does so in Down Syndrome fibroblasts [10]). The inhibition of mitochondrial function at 300 µM may be a direct effect of inhibition of Complex IV by HMPNSE (as demonstrated by our measurements); it is conceivable that this effect is related to a direct inhibitory action of HMPSNE on Complex IV.

The biochemical properties of 3-MST are such that in the intracellular environment it is more prone to the formation of polysulfides, than the formation of H_2_S [19]. Polysulfides are now increasingly recognized as biological signaling molecules; their properties are different from those of H_2_S. For instance, the sulfhydration of protein cysteines (Cys-SH to Cys-SSH), a posttranslational modification generally attributed to H_2_S, is the consequence of the action of biologically active polysulfides—and not a direct effect of H_2_S [49]. (Nevertheless, H_2_S can S-sulfurate Cys-SOH and Cys-SNO to Cys-SSH [49]). Post-translational modifications of various mitochondrial or extramitochondrial 3-MST-derived H_2_S are likely follow a different time-course and concentration-response than some of the other biological effects elicited by H_2_S. Indeed, metabolomic analysis of HMPSNE-treated endothelial cells revealed many alterations in various biochemical pathways, the molecular mechanism of which are not yet fully understood [45]. The multitude of effects of H_2_S and polysulfides on various cellular targets may also explain some of the apparently conflicting findings between bioenergetic and functional responses reported in the current article.

In the context of tumor biology, we must emphasize that the role of 3-MST must be viewed not only within the tumor cell itself, but also in the tumor microenvironment. 3-MST is an enzyme that is expressed in vascular structures (and, indeed, it regulates endothelial cell proliferation, migration and angiogenesis [45]). 3-MST is also likely to be present in various immune cells [50], although its functional role (in general, or in particular with respect to tumor immunology) remains to be clarified in future studies. 3-MST is also expressed in normal parenchymal cells, where it may be needed for various fundamental biological processes. When considering inhibiting 3-MST for potential antitumor effects in vivo, the net sum of all of these processes must be considered. Future studies are expected to address some of these issues.

## 5. Conclusions

The current studies identify 3-MST as the principal source of biologically active H_2_S in the murine colon cancer cell line CT26 and indicate that 3-MST-derived H_2_S is involved in the regulation of cell proliferation, migration and bioenergetics. We utilized the novel pharmacological 3-MST inhibitor HMPSNE and the present article is, in fact, one of the handful of recent studies [26,45,51] that utilizes this inhibitor (which, to date, appears to be the only 3-MST pharmacological inhibitor with sufficient potency and selectivity to enable cell-based studies). Using a live cell imaging assay, we have also directly demonstrated the inhibitory effect of HMPSNE on cellular H_2_S production. The current report also demonstrates that HMPSNE exerts a bell-shaped response on various bioenergetic parameters in CT26 cells. Since CT26 cells do not express CBS (another H_2_S-producing enzyme that contributes to the maintenance of various biological effects in various other forms of cancer), this cell line may serve as a useful tool to investigate the functional role of 3-MST in cancer cells in vitro and in vivo.
